# Assessment of Collaborative Problem Solving Based on Process Stream Data: A New Paradigm for Extracting Indicators and Modeling Dyad Data

**DOI:** 10.3389/fpsyg.2019.00369

**Published:** 2019-02-26

**Authors:** Jianlin Yuan, Yue Xiao, Hongyun Liu

**Affiliations:** ^1^Educational Science Research Institute, Hunan University, Changsha, Hunan, China; ^2^Faculty of Psychology, Beijing Normal University, Beijing, China; ^3^Beijing Key Laboratory of Applied Experimental Psychology, Faculty of Psychology, Beijing Normal University, Beijing, China

**Keywords:** collaborative problem solving, process stream data, indicator extracting, dyad data, multidimensional model

## Abstract

As one of the important 21st-century skills, collaborative problem solving (CPS) has aroused widespread concern in assessment. To measure this skill, two initiative approaches have been created: the human-to-human and human-to-agent modes. Between them, the human-to-human interaction is much closer to the real-world situation and its process stream data can reveal more details about the cognitive processes. The challenge for fully tapping into the information obtained from this mode is how to extract and model indicators from the data. However, the existing approaches have their limitations. In the present study, we proposed a new paradigm for extracting indicators and modeling the dyad data in the human-to-human mode. Specifically, both individual and group indicators were extracted from the data stream as evidence for demonstrating CPS skills. Afterward, a within-item multidimensional Rasch model was used to fit the dyad data. To validate the paradigm, we developed five online tasks following the asymmetric mechanism, one for practice and four for formal testing. Four hundred thirty-four Chinese students participated in the assessment and the online platform recorded their crucial actions with time stamps. The generated process stream data was handled with the proposed paradigm. Results showed that the model fitted well. The indicator parameter estimates and fitting indexes were acceptable, and students were well differentiated. In general, the new paradigm of extracting indicators and modeling the dyad data is feasible and valid in the human-to-human assessment of CPS. Finally, the limitations of the current study and further research directions are discussed.

## Introduction

In the field of education, some essential abilities named Key Competencies (Rychen and Salganik, 2003) or 21st Century Skills (Partnership for 21st Century Skills, 2009; Griffin et al., 2012) have been identified. Students must master these skills if they want to live a successful life in the future. Collaborative problem solving is one of the important 21st century skills. Since computers have substituted for workers to complete many explicitly rule-based tasks (Autor et al., 2003), non-routine problem-solving abilities and complex communication and social skills are becoming increasingly valuable in the labor market (National Research Council, 2011). This set of special skills can be generalized as the construct of Collaborative Problem Solving (Care and Griffin, 2017).

The importance of CPS has spurred researchers in the educational area to assess and teach the skill. However, effectively measuring CPS challenges the current assessment area (Wilson et al., 2012; Graesser et al., 2017, 2018). Because of the complexity of CPS, the traditional testing approaches, such as the paper-pencil test, are inappropriate for it. Therefore, two initiative approaches have been created and applied to the assessment of CPS (Scoular et al., 2017), which are the human-to-human mode and the human-to-agent mode. The human-to-human mode was created by the Assessment and Teaching of 21st Century Skills (ATC21S) project for measuring CPS (Griffin and Care, 2014). It requires two students to collaborate and communicate with each other to solve problems and achieve a common goal. A computer-based testing system has been developed to undisturbedly record students’ operation actions, such as chatting, clicking buttons, and dragging objectives, and to generate process stream data (also called log file data; Adams et al., 2015). ATC21S also puts forward a conceptual framework of CPS (Hesse et al., 2015), which includes social and cognitive components. The social component refers to the collaboration part of CPS and the cognitive component refers to the problem solving part. Within the social dimension, there are three strands that are participation, perspective taking, and social regulation. The cognitive dimension includes two strands, task regulation and learning and knowledge building. Each strand contains several elements or subskills, and a total of 18 elements are identified in the framework. Indicators mapped to the elements are extracted from the log file data, and then are used to estimate individual ability (Adams et al., 2015). The Programme for International Student Achievement (PISA) employed the human-to-agent mode for the CPS assessment in 2015 (OECD, 2017a). A computer-based testing system for it has been developed, where computer agents are designed to interact with test-takers. The agents can generate chat messages and perform actions, and test-takers need to make responses (Graesser et al., 2017; OECD, 2017b). These responses, like answers of traditional multiple-choice items, can be directly used to estimate individual CPS ability.

There are many discussions about which is the better way to assess CPS between the two approaches. ATC21S takes the view that the human-to-human interaction is more likely to yield a valid measure of collaboration while the human-to-agent interaction does not conform with the real-world situation (Griffin et al., 2015). Graesser et al. (2017) indicate that the human-to-agent mode provides consistency and control over the social collaboration and that thus it is more suitable for the large-scale assessment. Studies have also shown that each approach involves limitations and have suggested further research to find comprehensive conclusions (Rosen and Foltz, 2014; Scoular et al., 2017). However, from the perspective of data collection, process stream data generated by the human-to-human mode is a record of the whole process of students’ actions in computer-based assessment. Based on the data, researchers can reproduce the process of how students collaborate and solve problems, which provides insight into students’ cognitive processes and problem solving strategies. In addition, technological advance promotes researchers in assessment area to focus on the process of solving problems or completing tasks, not just the test results. For example, numerous studies of problem solving assessment took a procedural perspective with the assistance of some technology-based assessment systems (PIAAC Expert Group in Problem Solving in Technology-Rich Environments, 2009; Zoanetti, 2009; Greiff et al., 2013; OECD, 2013). These systems could collect the process data and record problem-solving results simultaneously. Thus, the assessment can reveal more about students’ thinking process. By comparison, responses of multiple-choice items in the human-to-agent mode can only provide limited information. Therefore, we choose the human-to-human mode in the current study.

However, process stream data cannot be directly used to estimate individual ability. The theory of Evidence-centered Design (ECD) indicates that measurement evidence must be identified from these complicated data before latent constructs are inferred (Mislevy et al., 2003). In the context of educational assessment, existing methods for identifying measurement evidence from process data can be classified into two types. One type is derived from the field of machine learning and data mining, such as Clustering and Classification (Herborn et al., 2017; Tóth et al., 2017), Natural Language Processing and Text Mining (He and von Davier, 2016; He et al., 2017), Graphic Network models (Vista et al., 2016; Zhu et al., 2016), and Bayesian Networks (Zoanetti, 2010; Almond et al., 2015). These data-driven approaches aggregate process data to detect specific behaviors or behavioral patterns that are related to problem-solving outcomes as measurement evidence. Another type of methods can be seen as the theory-driven behavior coding, which means that specific behaviors or behavioral patterns in process data are coded as indicators to demonstrate corresponding skills. This approach was adopted in the CPS assessment of ATC21S. ATC21S defined two categories of indicators: direct and inferred indicators (Adams et al., 2015). Direct indicators can be identified clearly, such as a particular action performed by a student. Inferred indicators are related to sequential actions that represent specific behavioral patterns (Adams et al., 2015). The presence or absence of particular actions or behavioral patterns is the direct evidence that can be used to infer students’ abilities. If a corresponding action or behavioral pattern exists in process stream data, the indicator is scored as 1. Otherwise, it is scored as 0. From the perspective of measurement, indicators play the role of traditional items for estimating individual ability.

The theory-driven behavior coding seems effective to obtain measurement evidence from process data, but there exists a problem, that is, how to extract indicators for the dyad members in the human-to-human assessment mode. The ATC21S project adopted the asymmetric mechanism as the basic principle for task design (Care et al., 2015), which is also called jigsaw (Aronson, 2002) or hidden-profiles (Sohrab et al., 2015) in other research. The asymmetric design means that different information and resources are assigned to the two students in the same group so as to facilitate collaborative activities between them. As a result, they will perform different actions during the process of completing tasks, such as different operations, chat messages, and work products, and will generate their unique process stream data. ATC21S only extracted the same indicators for the two students. This means that the unique information contained in each student’s process stream data is ignored, while this information can demonstrate individual skills. Therefore, a comprehensive strategy must be considered to address the complexity of indicator extracting.

Another important problem related to the human-to-human mode is the non-independence between the dyad partners (Griffin et al., 2015). In the ATC21S project, two unacquainted individuals are assigned to work on a common task together. Because of the asymmetric design, they need to exchange information, share resources, negotiate and manage possible conflicts, and cooperate with each other. Each individual member cannot progress through the tasks without his/her partner’s assistance. This kind of dependence is called the dyad relationship (Alexandrowicz, 2015). Therefore, a concerned issue is whether the dyad dependence would affect individual scores (Griffin et al., 2015). In the measurement, the dyad relationship violates the local independence assumption of the measurement model. The ATC21S project used the unidimensional Rasch model and the multidimensional Rasch model in calibration (Griffin et al., 2015), and neglected the dyad dependence. However, group assessment has caught the attention of researchers in the measurement field. New approaches and models have been proposed for effective measurement within group settings (von Davier, 2017). Methodologies, such as weighted analysis and multilevel models, were suggested to allow group dependence (Wilson et al., 2012). Wilson et al. (2017) utilized item response models with and without random group effect to model dyad data. Results indicated that the model with the group effect fit better (Wilson et al., 2017). Andrews et al. (2017) used the Andersen/Rasch (A/R) multivariate IRT model to explore the propensities of dyads who followed certain interaction patterns. Alexandrowicz (2015) proposed a multidimensional IRT model to analyze dyad data in social science, in which each individual member had their unique indicators. Researchers have also proposed several innovative statistical models, such as stochastic point process and Hawkes process, to analyze the dyadic interaction (Halpin and De Boeck, 2013; von Davier and Halpin, 2013; Halpin et al., 2017). Olsen et al. (2017) extended the additive factors model to account for the effect of collaboration in the cooperative learning setting. Besides, computational psychometrics that incorporates techniques from educational data mining and machine learning has been introduced into the measurement of CPS (von Davier, 2017). For example, Polyak et al. (2017) used Bayes’ rule and clustering analysis in real-time analysis and post-game analysis, respectively. However, there is no definite conclusion on how to model the dyad data.

### The Present Study

We agree with the view that the human-to-human interaction is more likely to reveal the complexity and authenticity of collaboration in the real world. Therefore, following the approach of ATC21S, this study employed the human-to-human mode in the assessment of CPS. Students were grouped in pairs to complete the same tasks. The asymmetric mechanism was adopted for task design. Particular actions or behavioral patterns were identified as observable indicators for inferring individual ability. Distinct from the ATC21S approach, we considered a new paradigm for extracting indicators and modeling the dyad data. The main work involved in this study can be classified into three parts.

(1) Following the asymmetric mechanism, we developed five tasks and integrated them into an online testing platform. Process stream data were generated by the platform when the test was going on.
(2) Because of the asymmetry of tasks, we hold that there are unique performances of each member in the dyad for demonstrating their individual skills. Therefore, we extracted individual indicators for each dyad member based on his/her unique process stream data. At the same time, we also identified group indicators that reflected the dyad’s contribution and wisdom.
(3) Based on the special design of indicators, we utilized a multidimensional IRT model to fit the dyad data, in which each dyad member was attached with their individual indicators and group indicators.

## Design and Data

### Conceptual Framework of CPS

The CPS framework proposed by ATC21S was adopted in this study, while its detailed description can be seen in Hesse et al. (2015). A total of 18 elements were identified. ATC21S has given a detailed illustration of each element, including its implication and different performance levels (Hesse et al., 2015). The specification provides full insight into the complex skills. More importantly, it serves as the criterion for identifying indicators in this study.

### Task Design and Development

We developed five tasks in the present study. To complete each task, two students needed to compose a group. These tasks were designed following the asymmetric mechanism. The two students would obtain different information and resources so they have to cooperate with each other. The current assessment was planned for 15-year-old students, and the problem scenarios of all tasks were related to students’ daily life. To illustrate the task design, one of these five tasks, named Exploring Air Conditioner, is presented in Appendix. This task was adapted from the task of Climate Control released by PISA2012 (OECD, 2012), which was applied to the assessment of individual problem solving in a computer-based interactive environment. We adapted it for the context of CPS assessment.

To capture students’ actions, we predefined a series of events for each task, which can be classified into two types: common and unique events. The common events refer to universal events that would happen in all collaborative assessment tasks, such as the start and the end of a task, chat messages. The unique events occur in specific tasks due to the nature of the behaviors and interactions elicited in these tasks (Adams et al., 2015). Table 1 presents examples of event specifications for the task of Exploring Air Conditioner. Each event is defined from four aspects, including the event name, the student who might trigger it, the record format, and the explanation for how to capture it. The event specification plays an important role in the computer-based interactive assessment. Firstly, the events represent the key actions and system variables. These actions provide insight into the cognitive process of performing the task. Secondly, the event specification provides a uniform format for recording students’ behaviors, which is beneficial to explain the process stream data.

**Table 1:** Examples of events defined in the task of Exploring Air Conditioner.

Event type	Event name	Role	Record format	Explanation of capturing an action
Common events	Task start	A, B	task start	Record the start of a task
	Task end	A, B	task end	Record the end of a task
	Chat	A, B	free-form chat messages	Record the content of chat messages
Unique events	Control A	A	controlA: *status*	Record the action of changing the position of the slider in the control A
	Control B	A	controlB: *status*	Record the action of changing the position of the slider in the control B
	Control C	B	controlC: *status*	Record the action of changing the position of the slider in the control C
	Control D	B	controlD: *status*	Record the action of changing the position of the slider in the control D
	Apply	A, B	apply: *A* or *B*	Record the action of clicking the button of Apply


Based on the design of problem scenarios and event specifications, the mainstream techniques of J2EE and MySQL database were adopted for implementing the five tasks. Besides, an online testing platform of multi-user architecture was developed for delivery of all tasks, providing convenience for user login, task navigation, and system administration. The development of tasks and the testing platform followed an iterative process of software development. With the mature platform, students’ actions with time stamps could be undisturbedly recorded into the MySQL database as the test progressed, thus the process stream data could be generated.

### Data Collection

#### Procedures

Before the test, we established a set of technical standards for the computer device and internet access to choose schools with perfect Information and Communication Technology (ICT) infrastructure. Since most students and teachers are unfamiliar with the web-based human-to-human assessment of CPS, a special procedure of test administration was considered in the present study. The whole testing process took 70 min, which was divided into two stages. The practice stage was about 10 min, during which examiners needed to illustrate to students what was the human-to-human assessment of CPS. Meanwhile, one task was used as an exercise to help students understand rules. After the practice, the other four tasks were used as assessment tasks in the formal test stage, and 60 min were assigned. Students were demanded to follow the test rules just like what they did in a traditional test, except that they needed to collaborate with their partners via the chat box. Examiners only provided technological assistance during the period. Student’s data generated in the four assessment tasks would be used for indicator extracting and subsequent data analysis.

#### Participants

Four hundred thirty-four students with an average age of approximately 15 years old participated in the assessment, including 294 students from urban schools and 140 students from rural schools in China. All students possess basic ICT skills, such as typing words, sending email, and browsing websites. Since the present study does not focus on the problem of team composition, all the students were randomly grouped in pairs and each student was assigned to a role (A or B) in the group. During the test, students would act as the same role and two members in the dyad group were anonymous to each other.

#### Ethics Statement

Before we conducted the test, the study was reviewed and approved by the research committee in Beijing Normal University, as well as by the committee in local government. The school teachers, students, and students’ parents had clear understanding about this project and how the data were collected. All the students were required to take the written informed consent form to their parents and ask their parents to sign it if they agreed with it.

#### Process Stream Data

**FIGURE 1:**
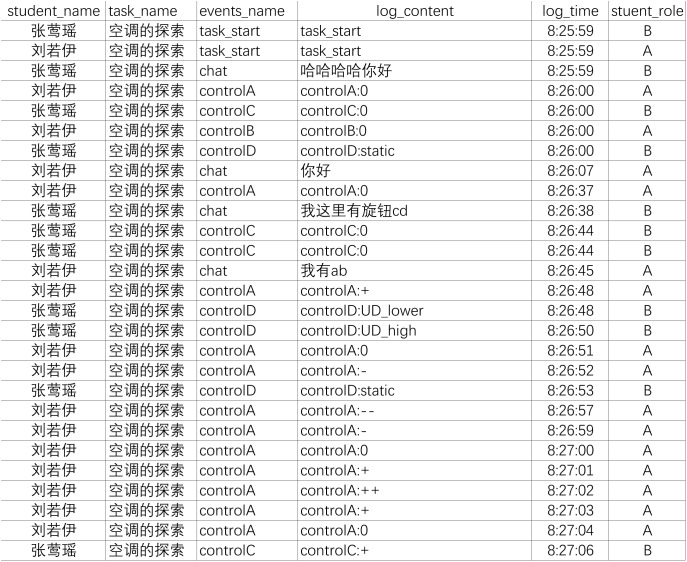
A part of process stream data from Exploring Air Conditioner.

As mentioned above, we predefined a series of events for each task, which represent specific actions and system variables. When the test was in progress, students’ actions with time stamps would be fully recorded into a database and then process stream data would be generated. Figure 1 presents a part of the process stream data from the task of Exploring Air Conditioner, which is exported from MySQL database. The process stream data is constituted by all the events generated by dyad members from the start to the end of tasks, including students’ actions, chat messages and status changes of system variables. Each event was recorded as a single row and tagged with the corresponding student identifier, the task identifier, the event content, the role of the actor in the dyad, and the time of the event.

### Data Processing

Data processing included two steps. First, indicators that serve as measurement evidence were identified and extracted from process stream data. This procedure is an analogy to item scoring in traditional tests. Second, to estimate individual ability precisely, we used a multidimensional Rasch model to fit the dyad data. The quality of indicators and the test was also evaluated in this stage.

## Indicator Extracting

### Rationale for Indicator Extracting

From the perspective of measurement, it is hard to directly judge the skill level of each student based on the process stream data. According to the theory of ECD (Mislevy et al., 2003), measurement evidence must be identified from process stream data for inferring latent ability. Since the abstract construct of CPS has been deconstructed into concrete elements or subskills, it is easier to find direct evidence for demonstrating these subskills or elements than the whole construct. To build up the reasoning chain from process stream data to assessment inference, a theoretical rationale has been commonly taken in many process-oriented assessments, which is that “students’ skills can be demonstrated through behaviors which are captured in the form of processes” (Vista et al., 2016). In other words, the observable features of performance data can be used to differentiate test-takers in high and low ability levels (Zoanetti and Griffin, 2017). If the rules of behavior coding that link the process data and inference are established, specific actions or sequential actions in process stream data can be coded into rule-based indicators for assessment (Zoanetti, 2010; Adams et al., 2015; Vista et al., 2016; Zoanetti and Griffin, 2017). This procedure is called indicator extracting in the current study.

In the present study, indicator extracting includes two steps. First, the theoretical specification of indicators was set up, which illustrates why each indicator can be identified and how to extract it. Second, all the indicators were evaluated by experts and the validated indicators were used to score process stream data. Thus, the scoring results of each student were obtained.

### Indicator Specification

Based on single events or sequential actions in process stream data, we defined both direct and inferred indicators mapped to elements of the CPS framework. The direct indicator could be clearly identified from a single event, such as the success or failure of a task and a correct or false response to a question. However, the inferred indicator identified from a sequence of actions must be rigorously evaluated. Table 2 outlines examples for illustrating the specifications of inferred indicators.

**Table 2:** Examples of indicator specifications.

Indicator name	Mapping element	Definition of the indicator	Algorithm	Output
T1A01	Action	The number of messages and actions generated by student A, reflecting his/her activeness in collaboration.	In the process stream data of student A, count all the events that he/she generated.	The count value.
T1G02	Interaction	The number of interactive chat blocks (A, B) between two students, reflecting their interaction. Consecutive chats without interrupted actions from the same student are counted as one. (e.g., A,B,A,B = 2 chat blocks; AA,B,A,B = 2 chat blocks)	Step 1: Find all sequences of consecutive chat messages without any interrupted actions in the process stream data of Student A and B.	The count value.
			Step 2: Count the number of chat blocks in each chat sequence. Add one to the value of the indicator if one chat block is found.	


As can be seen from Table 2, the specification of each indicator includes five aspects. First, all indicators were named following a coding rule. Taking the indicator ‘T1A01’ as an example, ‘T1’ represents the first task, ‘A’ represents that it is identified for student A and is an individual indicator (‘G’ represents a group indicator), and ‘01’ is a numerical code in the task. Then, the mapping element shows what element of CPS this indicator is related to. The definition provides a theoretical description of why it can be identified. The algorithm elaborates the detailed process of how to extract it from process stream data, which is the basis for developing the scoring program. In the last column of the table, the type of the scoring result is simply described. There are two types of output: the count value and the dichotomous value.

### A New Paradigm of Extracting Indicators

Distinct from ATC21S, we defined two types of indicators, group and individual indicators. The group indicators are used to illustrate the underlying skills of the two students as a dyad, reflecting the endeavor and contribution of the group. As the indicator T1G02 in Table 2, the interactive conversation cannot be completed by any individual member and it needs the two students’ participation. Another typical group indicator is identified from task outcomes, that is, the success or failure of each task. The individual indicators are used to demonstrate the underlying skills of the dyad members. Owing to the asymmetric task design, the two members in a group would take different and unique actions or sequential actions, which are used to identify these indicators.

### Indicator Validation and Scoring

We defined 8 group indicators and 44 individual indicators (23 for student A and 21 for student B) across the four assessment tasks. To reduce the errors of indicator specifications caused by subjective judgment, indicators were validated by means of expert evaluation. A five-member panel constituted by domain and measurement experts were consulted to evaluate all indicator specifications. Materials, including problem scenario designs, event definitions, samples of process stream data, and all indicator specifications, were provided to them. Experts were demanded to evaluate whether the indicator specifications were reasonable and to give suggestions for modification. An iterative process including evaluation and modification of indicator specifications was used. The process was repeated until all experts agreed on the modified version of all indicators.

Because it is unpractical to score process stream data of all students by human rating, an automatic scoring program was developed based on R language, according to the final specifications of all indicators. We randomly selected 15 groups (30 students) from the sample and obtained their scores separately by the scoring program and a trained human rater. The Kappa consistency coefficient determining the validation of the automatic scoring was calculated for each dichotomously scored indicator. For a few indicators with low Kappa values, we modified their scoring algorithm until their consistency was acceptable. The final results of Kappa consistency for all indicators were shown in Section “Indicator Validation Results.” We did not use the Kappa coefficient for indicators with count values, i.e., frequency-based indicators, since the coefficient was based on categorical data. Instead, the reliability of automatic scoring for these indicators were rigorously checked by the research team. The scoring results of each indicator, which were generated by the scoring program and the human rater, were compared based on the randomly selected data of 3 to 5 students. Once there were any differences, we modified the scoring algorithm until the automatic scoring results were the same as scores given by the human rater. After the validation, the process stream data of 434 participants were scored by the automatic scoring program.

### Conversion of Frequency-Based Indicators

**FIGURE 2:**
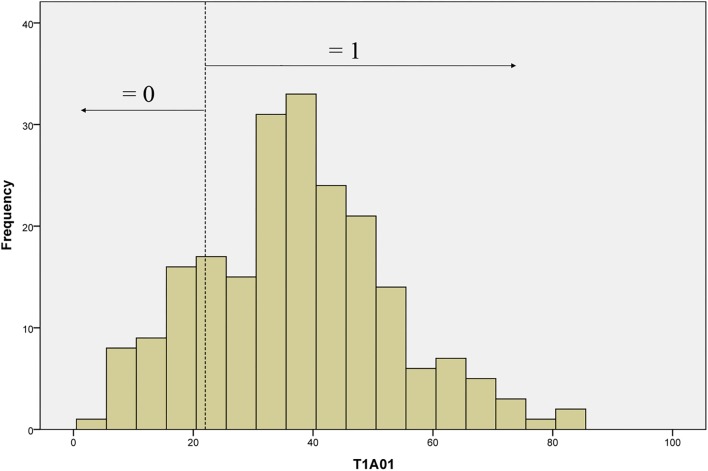
The frequency distribution of T1A01.

For model estimation, the count values of frequency-based indicators needed to be converted into discrete values. Since the unique nature of the scoring approach for process data, there is little existing literature that could be used as a guide for the conversion. ATC21S proposed several approaches (Adams et al., 2015), and two of them were adopted in the study. Specifically, we did the transformation by setting thresholds according to the empirical frequency distribution or the meaning of count values. First, some indicators were converted by setting cut-off values according to their distributions from empirical data. For instance, the frequency distribution of T1A01 (the first indicator in Table 2), as shown in Figure 2, had a mean of 37.18 and a standard deviation of 15.74. This indicator was mapped to the element of Action in CPS framework and evaluated student activeness in the task. Obviously, a more active student would generate more behaviors and chats. Following the approach of ATC21S (Adams et al., 2015), the cut-off value was set at 22, to which the mean minus a standard deviation (21.44) was rounded up. Thus, students whose number of behaviors and chats less than 22 (*n* < 22) got a score of 0, while those with the number more than 22 (*n* ≥ 22) got a score of 1. Second, some frequency-based indicators only contain limited values and each count value was easily interpretable. Thus, a particular value with special meaning could be set as the threshold to transform the indicator. Based on the two approaches, all frequency-based indicators were converted to dichotomous or polytomous variables. Then, all indicators could serve as evidence in the measurement model for inferring students’ ability.

## Modeling Dyad Data

### Model Definition

In the human-to-human assessment mode of CPS, two students in the same group establish a dyad relationship; hence we call the scoring results dyad data. As mentioned above, how to model the dyad data is a central concern in the assessment of CPS (Wilson et al., 2012; Griffin et al., 2015). Researchers have proposed a number of models to account for the non-independence between the dyad members, such as the multilevel IRT models (Wilson et al., 2017), Hawkes process (Halpin and De Boeck, 2013), and the multidimensional IRT models (Alexandrowicz, 2015). Since group and individual indicators were simultaneously extracted in this study, we employed a multidimensional IRT model to fit the dyad data. The multidimensional model is the extension of the unidimensional model when more than one latent trait is assumed to exist in a test. Some researchers have employed multidimensional IRT models to fit dyad data (Alexandrowicz, 2015). This enlightened us to apply the multidimensional model to the human-to-human assessment of CPS, where two members in a dyad are regarded as two different dimensions.

**FIGURE 3:**
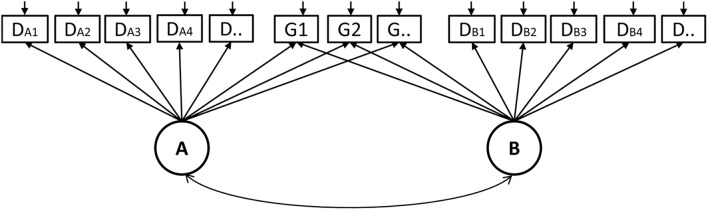
A diagram of the within-item Rasch model for the dyad data.

There are two types of multidimensional models: within-item and between-item multidimensional models (Adams et al., 1997). In this study, we chose the within-item multidimensional Rasch model for the dyad data. As depicted in Figure 3, student A and B are regarded as two dimensions, where the latent factor A and B, respectively represent the CPS ability of the role A and B. The indicator D_A1_, D_A2_, …, attached to factor A, are individual indicators of student A. Similarly, D_B1_, D_B2_, …, are individual indicators for student B. The indicator G1, G2, …, are group indicators that are simultaneously attached to factor A and B. Specifically, the Multidimensional Random Coefficients Multinomial Logit Model (MRCMLM; Adams et al., 1997) was adopted to fit the data and its formula is

(1) $$P(X_{ik}=1;A,B,\xi |\theta )=\frac{\exp\left(b_{ik}\theta +{\alpha^{\prime }}_{ik}\xi \right)}{\Sigma_{k=1}^{K_{i}}\exp\left(b_{ik}\theta +{\alpha^{\prime }}_{ik}\xi \right)}$$

where θ is a vector representing the person’s location in a multidimensional space and is equal to (θ*_A_*,θ*_B_*) in the current study. The notations of A, B, and *ξ* represent the design matrix, the scoring matrix, and the indicator parameter vector, respectively. X*_ik_* = 1 represents a response in the *k*th category of indicator *i*. The design matrix *A* is expressed as


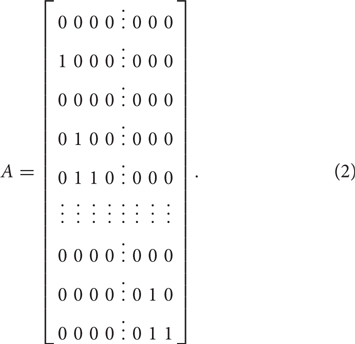


where each row corresponds to a category of an indicator and each column represents an indicator parameter. For example, indicator 1 and 2 have two and three categories respectively, which correspond to the first to second row and the third to fifth row in the above matrix. The scoring matrix *B* specifies how the individual and group indicators were attached to dimension θ*_A_* and θ*_B_*, which is expressed as


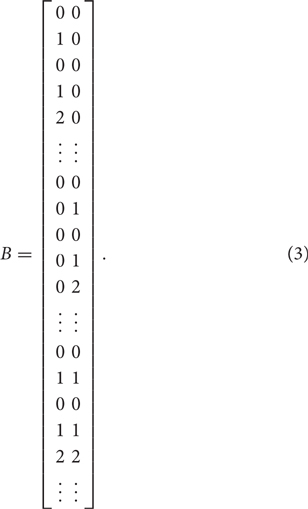


where each row corresponds to a category of an indicator and each column denotes a dimension. In the above matrix *B*, for example, the first five rows denote that the first indicator (scored as 0 or 1) and the second indicator (scored as 0, 1, or 2) are individual indicators scored on the first dimension (θ*_A_*). The middle several rows correspond to those individual indicators scoring on the second dimension (θ*_B_*). The last several rows indicate those indicators measuring both dimensions, i.e., group indicators.

### Calibration

Indicator calibration was performed by ConQuest 3.0, which included two stages. At the first stage, all the indicators (44 individual indicators and 8 group indicators) were calibrated with the one-parameter multidimensional Rasch model. Since the Rasch model only provides difficulty estimates, indicator discrimination was calculated by the traditional CTT (Classical Testing Theory) method in ConQuest. To evaluate the indicator quality, we used some important indicator indexes, such as discrimination, difficulty, and Infit mean square (Information Weighted Mean Squared residual goodness of fit statistic, often represented as MNSQ). In addition, researchers suggested special sequential actions in the process of problem solving were related to task performance (He and von Davier, 2016). This enlightened us to use the correlation between procedural indicator and the corresponding task outcome as a criterion for evaluating indicator quality. It was assumed that good procedural performance is always associated with a better outcome. After comprehensive consideration, the indicators, of which the MNSQ outside the range of 0.77 and 1.33, the discrimination and correlation below zero, were excluded from the subsequent analysis. In the second stage, the selected indicators were used to estimate individual ability. Model fit indexes, indicator parameter estimates, and the case distribution based on these indicators provided by ConQuest were used to evaluate test quality.

## Results

Calibration is an exploratory process when it is carried out in test development. For saving space, here we only present the results in the second stage of calibration, which provides the final evidence for the test quality. A total number of 36 individual indicators and 8 group indicators were calibrated in the second stage. The results of calibration and indicator validation are as follows.

### Indicator Validation Results

The interrater reliability of twenty dichotomously scored indicators were validated by computing the Kappa consistency between the scoring program and the human rater. The results are shown in Table 3. According to the magnitude guideline, the consistency was excellent with a Kappa value over 0.75 and was fair to good with the value from 0.4 to 0.75 (Fleiss et al., 2013). As seen in Table 3, all indicators’ Kappa value are over 0.4 and there are 12 indicators with excellent Kappa consistency, indicating the reliability of automatic scoring.

**Table 3:** Kappa consistency of indicators between the scoring program and the human rater.

Indicators	Kappa coefficient	Indicators	Kappa coefficient	Indicators	Kappa coefficient
T1A03	0.659	T4A02	0.605	T4B03	0.852
T1A07	0.595	T1B03	1.000	T4B01	0.474
T1A09	0.857	T1B07	0.842	T1G01	0.857
T2A01	0.857	T1B09	0.471	T2G01	1.000
T3A04	0.587	T2B01	1.000	T3G01	0.789
T3A06	0.706	T3B04	1.000	T4G01	1.000
T4A03	0.700	T3B06	0.400		


### Model Fit

Model fit results are shown in Table 4. The sample size is the number of dyad groups, indicating a total number of 217 groups (434 students) participated in the assessment. Separation reliability describes how well the indicator parameters are separated (Wu et al., 2007), and the value of 0.981 indicates an excellent performance of test reliability. Dimension 1 and 2, respectively represent student A and B. Reliability of dimensions represents the degree of person separation. The value of 0.886 and 0.891 indicate that the test is sensitive enough to distinguish students at high and low ability levels. Wright and Masters (1982) showed that the indicator separation index and person separation index could be respectively used as an index of construct validity and criterion validity. Therefore, the results in the present study indicate the adequate validity of the test. The dimension correlation is calculated by estimated scores of student A and B, and the value of 0.561 indicates that dyad members are dependent on each other to a certain extent.

**Table 4:** Model fit of the two-dimensional Rasch model.

Sample size	Final deviance	Separation reliability	Reliability of dimension 1	Reliability of dimension 2	Correlation of dimension 1 and 2
217	12869.646	0.981	0.886	0.891	0.561


### Indicator Parameter Estimates and Fit

Indicator parameter estimates and fit indexes are presented in Table 5. The indicator difficulty estimates are within the range of -2.0 to 1.156 and have an average value of -0.107. Indicator discrimination, calculated by traditional CTT item analysis, falls within the range from 0.22 to 0.51 for most indicators. The MNSQ estimates and confidence interval are reported with *T-*value, and the accepted value of MNSQ ranges from 0.77 to 1.33 (Griffin et al., 2015). The MNSQ values of most indicators fall inside their confidence intervals and the absolute values of their corresponding T statistics are smaller than 2.0. As can be seen, the MNSQ of all indicators are reasonable and has an average value of 1.0, indicating good indicator fit.

**Table 5:** Results of indicator parameter estimates and fit.

Indicator	Discrimination	Difficulty	Error	MNSQ	Confidence interval		T
T1A01	0.24	-1.554	0.180	1.01	0.79	1.21	0.1
T1A04	0.34	-0.309	0.083	1.09	0.87	1.13	1.3
T1A07	0.42	0.139	0.142	0.95	0.93	1.07	-1.5
T1A09	0.26	1.189	0.163	0.99	0.84	1.16	-0.1
T2A01	0.46	-1.860	0.196	0.93	0.74	1.26	-0.5
T2A03	0.28	1.516	0.176	0.97	0.80	1.20	-0.3
T2A01	0.28	-0.723	0.149	0.99	0.89	1.11	-0.2
T3A01	0.39	-1.943	0.201	0.94	0.73	1.27	-0.4
T3A04	0.35	0.935	0.154	0.94	0.87	1.13	-0.9
T3A06	0.22	0.835	0.151	1.01	0.88	1.12	0.1
T3A01	0.15	-1.243	0.120	1.09	0.84	1.16	1.1
T3A02	0.15	0.048	0.084	1.30	0.88	1.12	4.5
T3A03	0.41	1.027	0.090	0.95	0.79	1.21	-0.4
T4A01	0.30	-1.681	0.190	0.98	0.76	1.24	-0.1
T4A03	0.50	0.655	0.151	0.88	0.90	1.10	-2.3
T4A04	0.38	0.794	0.154	0.92	0.88	1.12	-1.3
T4A01	0.44	0.408	0.116	0.94	0.83	1.17	-0.7
T4A02	0.39	0.412	0.147	0.94	0.91	1.09	-1.3
T4A03	0.49	-0.179	0.121	0.89	0.82	1.18	-1.3
T1B01	0.33	-1.781	0.193	0.95	0.75	1.25	-0.4
T1B04	0.25	-0.067	0.079	1.18	0.88	1.12	2.9
T1B07	0.31	0.077	0.141	0.99	0.93	1.07	-0.3
T1B09	0.36	1.025	0.157	0.96	0.86	1.14	-0.6
T2B01	0.39	-1.566	0.179	0.95	0.78	1.22	-0.4
T2B03	0.31	1.038	0.157	0.97	0.86	1.14	-0.4
T2B01	0.32	-0.896	0.152	0.96	0.87	1.13	-0.6
T3B01	0.36	-2.009	0.206	0.95	0.71	1.29	-0.3
T3B04	0.29	1.172	0.161	0.96	0.85	1.15	-0.5
T3B06	0.30	0.636	0.146	1.00	0.90	1.10	0.0
T3B01	0.22	-0.708	0.101	1.12	0.85	1.15	1.5
T3B02	0.04	0.036	0.085	1.34	0.88	1.12	5.0
T3B03	0.42	1.113	0.087	0.97	0.79	1.21	-0.3
T4B01	0.43	-1.748	0.193	0.96	0.75	1.25	-0.3
T4B03	0.37	0.329	0.145	0.96	0.92	1.08	-1.0
T4B01	0.22	-0.147	0.144	0.87	0.93	1.07	-3.8
T4B03	0.04	0.679	0.092	0.88	0.84	1.16	-1.5
T1G01	0.42	-0.277	0.080	1.18	0.87	1.13	2.6
T1G02	0.43	-0.035	0.076	1.18	0.88	1.12	2.9
T2G01	0.37	-0.439	0.043	1.10	0.82	1.18	1.1
T2G02	0.22	0.322	0.083	0.90	0.87	1.13	-1.4
T3G01	0.04	0.015	0.064	1.05	0.82	1.18	0.6
T3G02	0.50	0.163	0.061	1.07	0.83	1.17	0.8
T4G01	0.51	-0.157	0.070	1.01	0.81	1.19	0.1
T4G02	0.47	0.055	0.067	1.12	0.82	1.18	1.2


*In Conquest, discrimination is the product moment correlation between the case scores on the indicator and the corresponding case raw scores.*

### Indicator and Latent Distribution

**FIGURE 4:**
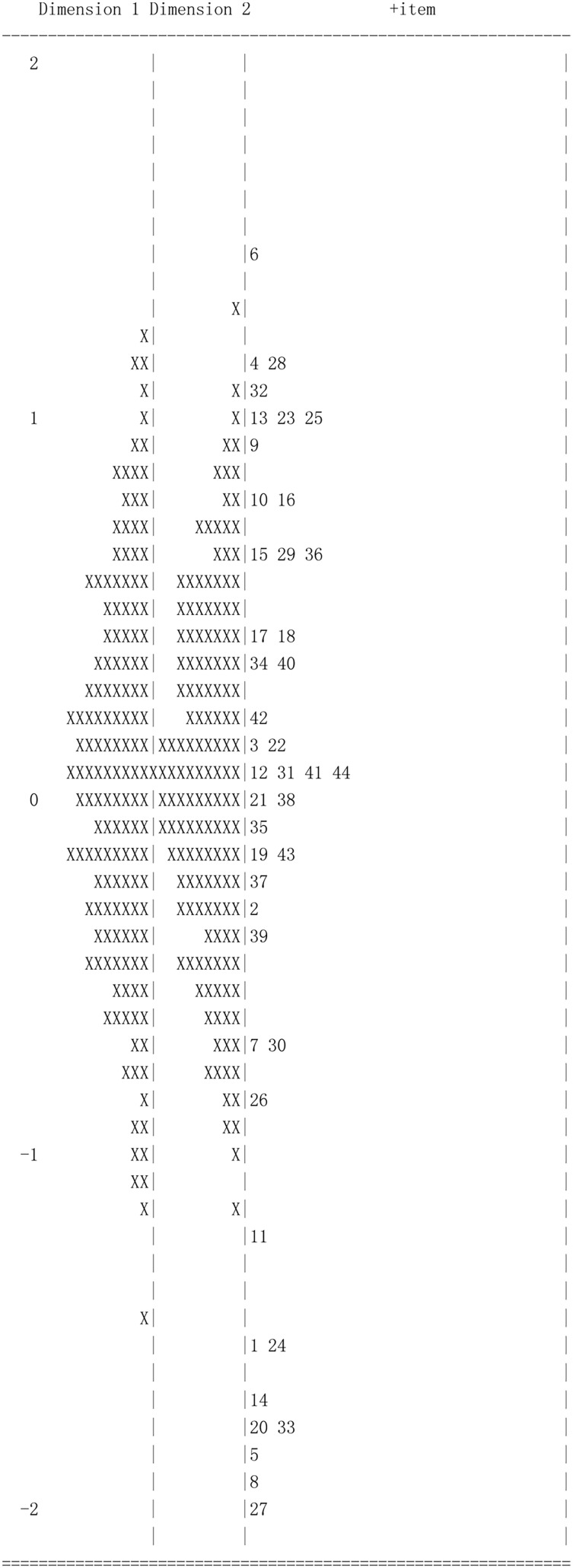
The indicator and latent distribution map of two-dimensional Rasch model. Each ‘X’ represents 1.4 cases.

ConQuest can output indicator and case distribution, in which the indicator difficulty and the student ability are mapped to the same logit scale. Figure 4 presents the distribution of indicator difficulty and student ability in the second stage of calibration. Dimension 1 and 2, respectively represent student A and B. Since the mean of latent ability is constrained as zero in ConQuest, students’ abilities are concentrated in the zero point of logit scale and approximate a Gaussian distribution. On the right of the map, indicators are dispersedly distributed from easy to difficult. There are 8 indicators whose difficulty parameters are below the lowest level of ability, indicating they were very easy for all students.

### Descriptive Analysis of Testing Results

Of the 434 participants, the minimum and maximum score respectively are -2.17 logits and 2.15 logits. Student ability estimates vary in the full range of 4.319 with a standard deviation of 0.68, indicating that students were well differentiated by the current assessment. Table 6 presents the descriptive statistics of students’ ability of successful group and failure group in each task. There are more students who successfully completed task 1 and 2 than those who failed, while the case is opposite for task 3 and 4. To some extent, this indicates the latter two tasks may be more difficult than the former two tasks. In addition, in all tasks, the mean ability of the students who successfully completed the task is higher than that of the unfinished students. It is consistent with common sense, indicating students’ ability estimation is reliable.

**Table 6:** Descriptive statistics of students’ ability of successful and failure group in each task.

	N	Minimum	Maximum	Mean	Std. deviation
Failure group in task 1	156	-2.169	1.183	-0.237	0.626
Successful group in task 1	254	-2.029	2.149	0.184	0.647
Failure group in task 2	210	-2.169	1.521	-0.205	0.629
Successful group in task 2	222	-1.975	2.149	0.181	0.676
Failure group in task 3	352	-2.170	1.900	-0.139	0.636
Successful group in task 3	82	-0.784	2.149	0.572	0.549
Failure group in task 4	226	-2.169	1.267	-0.210	0.585
Successful group in task 4	148	-1.150	2.150	0.485	0.552


## Discussion

The current study employed a human-to-human interaction approach initiated by the ATC21S project to measure the collaborative problem solving construct. Following the asymmetric mechanism, we designed and developed five tasks which two students need to partner with each other to work through. Moreover, we integrated the tasks into an online testing platform. There are several reasons impelling us to adopt the human-to-human interaction in the CPS assessment. One advantage is that it approximates to the situation in real life (Griffin et al., 2015) because it requires the real people to collaborate with each other and provides an open environment, such as a free-form chat box, for them to communicate. More importantly, the process stream data obtained provide informative insights into the process of collaboration and problem solving.

The task design is crucial in the present study, which includes the problem scenario design and the definition of events. The problem scenario design aims to elicit students’ latent ability of CPS effectively. Therefore, we adopted the asymmetric mechanism for it, which required dyad members to pool their knowledge and resources to achieve a common goal. The event definition is about how to record students’ actions in the process stream data. To solve it, we predefined a number of crucial events that represent key actions and system variables for each task. They are indispensable observations for understanding the process of performing tasks and provide a uniform format for recording the data stream. In addition, the technical architecture of tasks and the testing platform are important for developing a stable test system according to our experience, especially a well-constructed multi-user synchronization mechanism.

To tap the rich information from the process stream data, we need to identify indicators that could be mapped to the elements of the conceptual framework as measurement evidence. It has been found that particular sequential actions could be used as rule-based indicators for assessment (Zoanetti, 2010; Adams et al., 2015; Vista et al., 2016). Therefore, we identified specific actions or sequential actions as markers of complex problem solving process in the current study. However, distinct from the ATC21S approach, we defined two kinds of indicators, individual and group indicators, which reflect the underlying skills of individuals and groups, respectively. Owing to the asymmetry of resources, two members in a dyad would perform differently and generate unique process stream data, while their group performance would also be recorded. Therefore, we could investigate the CPS ability at both the individual and group level.

Another problem concerned by the present study is how to model the dyad data. ATC21S extracted the same indicators for dyad members and the dyad data was modeled by traditional methods. Hence, the local independence assumption of the measurement model was violated. We adopted the two-dimensional within-item Rash model to analyze the dyad data based on the new paradigm of indicator extracting, taking the dyad dependence into account. Results indicated that the model fit well and that indicator parameters and participants were separated well. All the indicator parameter estimates and indicator fit indexes were also reasonable and acceptable. Along with the logit scale, indicators were dispersedly distributed from easy to difficult. In general, the results of data analysis demonstrate that the new paradigm of extracting indicators and modeling the dyad data is a feasible method for CPS assessment.

## Limitation

As a tentative practice of CPS assessment, the current study also has some limitations. First, most indicators identified in the study are based on the events of operation actions, while students’ chat messages are not utilized effectively. Chatting is the only way for the two students to communicate in the human-to-human interaction. Thus, the messages contain abundant information that can be used as measurement evidence. However, extracting indicators from chats requires the technique of semantic analysis. We did not do that work due to our limitation of Chinese semantic analysis. Second, for some elements in the conceptual framework, such as audience awareness and transactive memory, there are no indicators that can be mapped. This is because it is unable to find corresponding sequential actions from process stream data. It is necessary to extract more indicators to ensure an effective measurement of CPS. Third, following the ATC21S’ approach, we set up cut-off values for frequency-based indicators based on their distributions of empirical data. This choice of thresholds is tentative and further research is needed for setting more accurate values. Fourth, in the present study, we randomly assigned participants into dyad groups and did not consider group composition, because the current work focuses on how to extract indicators and model the dyad data. However, it is obvious that the group composition would affect the process and results of the collaboration. Further research can consider employing advanced techniques to extract more reliable indicators or exploring the strategies for student grouping.

## Author Contributions

JY contributed to task design, scoring, data analysis, manuscript writing, and revision. HL contributed to organizing the study and manuscript revision. YX contributed to data collection and manuscript revision.

## Conflict of Interest Statement

The authors declare that the research was conducted in the absence of any commercial or financial relationships that could be construed as a potential conflict of interest.

## Figures and Tables

**FIGURE A1:**
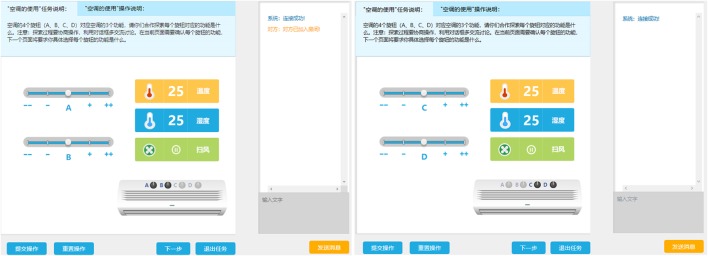
Screenshots of first pages in Exploring Air Conditioner.

## Appendix 1: The Task Description Of Exploring Air Conditioner

The interface of each task is in a unified form as shown in Figure A1. For each student, an instruction is presented at the top of the page to describe the problem scenario. A chat box for communication is in the right panel. Navigation buttons are placed at the bottom.

The task of Exploring Air Conditioner includes two pages. Figure A1 shows the first pages seen by the two students, respectively in the task. There are four controls on an air conditioner, which correspond to the regulation of temperature, humidity, and swing. Two students are demanded to explore the function of each control together. On the first page, student A can operate control A and B, and student B can operate control C and D. The function panel showing the temperature, humidity, and swing levels is shared by two students, which means the function status is simultaneously affected by two students’ operations. To complete the task, they have to exchange information, negotiate strategies for problem solving and coordinate their operations. But above all, they must follow the rule that “change one condition at a time.” After figuring out each control’s function, they can use the navigation button to jump to the next page where students need to submit their exploration results.
